# First detection of bee viruses in hoverfly (syrphid) pollinators

**DOI:** 10.1098/rsbl.2018.0001

**Published:** 2018-02-28

**Authors:** Emily J. Bailes, Kaitlin R. Deutsch, Judit Bagi, Lucila Rondissone, Mark J. F. Brown, Owen T. Lewis

**Affiliations:** 1 School of Biological Sciences, Royal Holloway University of London, Egham TW20 0EX, UK; 2 School of Geography and the Environment, University of Oxford, South Parks Road, Oxford OX1 3QY, UK; 3 Department of Zoology, University of Oxford, South Parks Road, Oxford OX1 3PS, UK; 4 Department of Entomology, Cornell University, Ithaca, NY 14853, USA

**Keywords:** *Varroa destructor* virus, acute bee paralysis virus, slow bee paralysis virus, emerging infectious diseases, honeybee, hover fly

## Abstract

Global declines of insect pollinators jeopardize the delivery of pollination services in both agricultural and natural ecosystems. The importance of infectious diseases has been documented in honeybees, but there is little information on the extent to which these diseases are shared with other pollinator orders. Here, we establish for the first time the presence of three important bee viruses in hoverfly pollinators (Diptera: Syrphidae): black queen cell virus (BQCV), sacbrood virus (SBV) and deformed wing virus strain B (DWV-B). These viruses were detected in two *Eristalis* species, which are behavioural and morphological bee mimics and share a foraging niche with honeybees. Nucleotide sequences of viruses isolated from the *Eristalis* species and *Apis mellifera* were up to 99 and 100% identical for the two viruses, suggesting that these pathogens are being shared freely between bees and hoverflies. Interestingly, while replicative intermediates (negative strand virus) were not detected in the hoverflies, viral titres of SBV were similar to those found in *A. mellifera*. These results suggest that syrphid pollinators may play an important but previously unexplored role in pollinator disease dynamics.

## 1. Introduction

Emerging infectious diseases (EIDs) are a global problem for biodiversity and human health [1]. Their occurrence has been associated with anthropogenic pressures, such as the global transport of managed animals and plants [1,2], which introduce diseases into novel hosts and alter natural disease dynamics [3]. EIDs can be particularly problematic for small and declining populations where ‘spillover’ from large managed populations can occur repeatedly, potentially resulting in the eventual extinction of the native population [3].

The positive-stranded RNA viruses found in managed honeybees (*Apis mellifera* and *Apis ceranae*) represent a key complex of potential EIDs that are shared with other wild bee pollinators [4,5]. These viruses have been implicated in the declines of wild bee populations, leading to concern for the economic and ecological value of associated ecosystem services [6,7]. Viruses originally thought to be honeybee-specific are now known to occur in and infect a wide range of wild bee species [8]. Interspecific transfer of these viruses, and other parasites, is thought to occur when individuals forage at the same flowers [4,9,10]. While many other taxa commonly share floral resources with bees, information on the presence of these diseases in taxa other than bees is poor [11]. To understand and manage disease pressure on pollinator populations, the role played by other taxa of flower visitors in the transmission of ‘bee’ viruses needs to be evaluated.

Hoverflies (Diptera: Syrphidae) regularly share flowers with bees and are important providers of pollination services [12,13]. Here, we investigate whether four abundant taxa of hoverflies act as hosts or potential vectors for six common bee viruses.

## 2. Material and methods

### (a) Sample collection

During 16–22 July 2016, 20 individuals each of honeybees and four of the most common UK species of hoverfly (*Episyrphus balteatus* (De Geer, 1776), *Platycheirus albimanus* (Fabricius, 1781), *Eristalis tenax* (Linnaeus, 1758) and *Eristalis arbustorum* (Linnaeus, 1758)) were collected with permission from grassland and open woodland habitats at Wytham Woods, Oxfordshire, UK (51.77°N, −1.33°W). Flies were identified while alive, then killed and stored at −80°C.

### (b) Molecular analysis

Total RNA was extracted from bee and hoverfly abdomens using a Direct-zol™ RNA MiniPrep kit (Zymo Research). cDNA was synthesized from 2 µg of the RNA using M-MLV Reverse Transcriptase (Promega) with 0.5 µg random hexamers (Invitrogen). Further details are given in the electronic supplementary material.

The presence or the absence of six common bee viruses (acute bee paralysis virus, ABPV; black queen cell virus, BQCV; deformed wing virus strain A, DWV-A, and strain B, DWV-B; slow bee paralysis virus, SBPV; sacbrood virus, SBV) was determined by RT-PCR (electronic supplementary material, primers in table S1). Positive samples identified by the amplification of the correct-sized product were verified by amplification in an independent RT-PCR reaction and subsequent Sanger sequencing (by Source Bioscience, Cambridge) to confirm they mapped to the virus of interest in the National Center for Biotechnology Information (NCBI) database. All amplicons of the correct size showed high sequence identity to the virus of interest (electronic supplementary material, table S2). All sequences are available at NCBI Genbank with the accession numbers MG737448–MG737473.

Viral titres of SBV and BQCV were quantified using qRT-PCR (see electronic supplementary material, primers in Table S1). To detect the negative strand of SBV and BQCV, which is indicative of virus replication, the protocol of de Miranda *et al*. ([14]; section 10.2.8.1) was followed using Superscript III (Invitrogen). A combined exonuclease and restriction digest was carried out on tagged cDNA to reduce the chance of false-positives and non-specific priming during PCR (electronic supplementary material).

### (c) Statistical analyses

Analyses were carried out in R v. 3.4.1 [15]. Viral titres were compared between *Apis* and hoverflies using Welch's *t*-tests following log-transformation. To compare virus incidence among species, we used *χ*^2^-tests in the coin package [16]. An approximated null distribution using 9999 replicate Monte Carlo simulations was used to account for zero/low counts.

## 3. Results

### (a) Detection of bee viruses by RT-PCR

**Figure 1.**
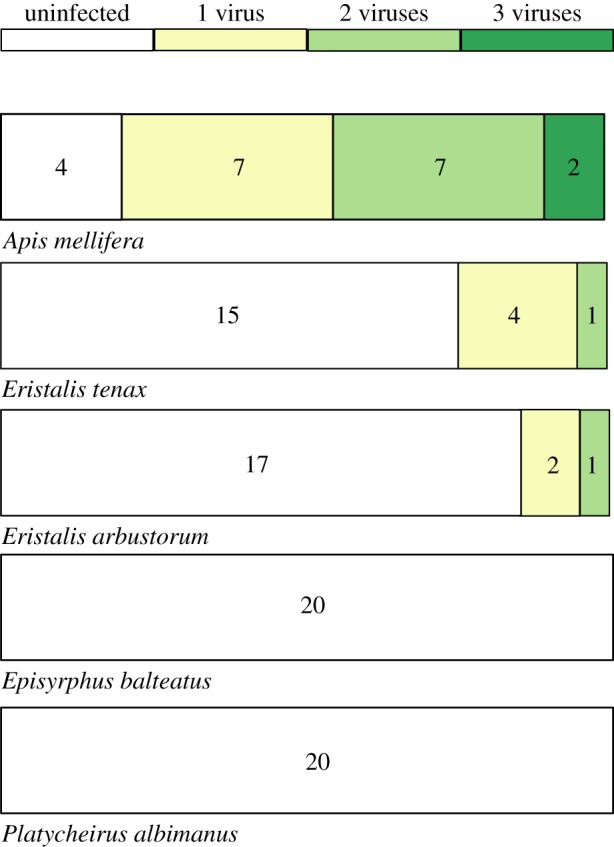
The number of viruses detected within an individual for each species. Bar width represents proportion of samples, numbers on bars are number of individuals. (Online version in colour.)

Viruses were detected in both *A. mellifera* and hoverflies (table 1 and figure 1). When considering positive results verified by independent amplification and sequencing (electronic supplementary material results), the most commonly detected virus in our samples was BQCV. BQCV was detected significantly more frequently in *A. mellifera* samples (13/20 samples) than in the hoverfly samples, *Er. tenax* (2/20) and *Er. arbustorum* (2/20; approximate test for differences among species: *χ*^2^ = 42.2, *p* < 0.001). BQCV was not detected in *P. albimanus* or *Ep. balteatus,* but there was no evidence that the proportion of samples with BQCV differed significantly among hoverfly species (*χ*^2^ = 4.2, *p* = 0.32). SBV was also frequently detected in *A. mellifera* (6/20), *Er. tenax* (4/20) and *Er. arbustorum* (1/20), but not in *P. albimanus* or *Ep. balteatus*. There was a significant difference in the proportion of SBV-positive samples across all species (*χ*^2^ = 14.7, *p* = 0.007), and across hoverfly species (*χ*^2^ = 19.2, *p* = 0.05).

**Table 1.** The number of individuals for each species where virus was verified to be present by RT-PCR.

species	BQCV	ABPV	SBV	SBPV	DWV-B	*n*
*Apis mellifera*	13	1	6	0	7	20
*Eristalis tenax*	2	0	4	0	0	20
*Eristalis arbustorum*	2	0	1	0	1	20
*Episyrphus balteatus*	0	0	0	0	0	20
*Platycheirus albimanus*	0	0	0	0	0	20

When assaying for the DWV complex, results from hoverfly samples were highly inconsistent for most sets of primers, and we were unable to verify the presence of DWV-A in our samples using two different primer sets (see electronic supplementary material; figure S1). DWV-B results were also difficult to verify, so detection of this virus in only one hoverfly sample may underestimate its true incidence.

### (b) Variation in BQCV and SBV sequences

Analysis of a 345 bp section of SBV capsid gene from *A. mellifera* and hoverfly sequences indicated that the strains of virus present in these individuals were highly similar (ranging from 95 to 99% nucleotide identity between hoverfly sequences and *A. mellifera* sequences; electronic supplementary material, table S3). Similarly, analysis of a 696 bp section of BQCV RNA-dependent RNA polymerase gene from *A. mellifera* and hoverfly sequences indicated high virus similarity (87–100% nucleotide identity of hoverfly sequences to *A. mellifera* sequences; electronic supplementary material, table S4).

### (c) Viral titres of BQCV and SBV

**Figure 2.**
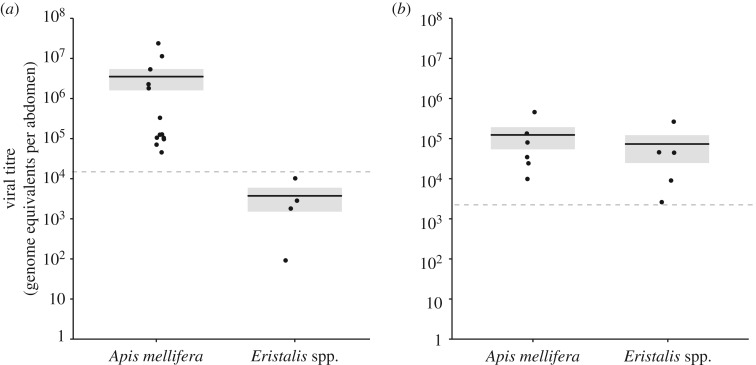
The viral titres (grey boxes represent s.e.; black line = mean) of honeybee and hoverfly abdomens. The dotted line represents the limit of the standard curve. Filled circles are individual data points. (*a*) BQCV titres; extrapolated for hoverflies (*b*) SBV titres.

For BQCV, *A. mellifera* samples contained 3.7 × 10^6^ ± 2.1 × 10^6^ genome equivalents per abdomen (mean ± s.e.; *n* = 13). This was significantly higher than in hoverflies (*t*_5.4_ = 5.0, *p* = 0.003), where all samples fell outside of our standard curve (a threshold equivalent to roughly 1.6 × 10^4^ viral equivalents per sample) but were extrapolated to contain 3.9 × 10^3^ ± 2.3 × 10^3^ genome equivalents per abdomen (*n* = 4; figure 2). For SBV, viral titres were not significantly different across *A. mellifera* and hoverfly samples (*t*_7.5_ = 0.8, *p* = 0.43), at 1.3 × 10^5^ ± 7.1 × 10^4^ (*n* = 6) and 7.4 × 10^4^ ± 5.0 × 10^4^ (*n* = 5) per abdomen, respectively.

### (d) Evidence of replication of BQCV and SBV

Negative strand-specific RT-PCR of BQCV- and SBV-positive samples indicated possible replication of BQCV in 2/13 *A. mellifera* workers and replication of SBV in 3/6 *A. mellifera* workers. Replication intermediates of SBV or BQCV were not detected in any hoverfly samples (five and four individuals, respectively), suggesting lack of viral replication.

## 4. Discussion

Our study is the first to detect bee viruses in hoverfly pollinators. By contrast, an earlier study found no evidence for the presence of DWV in four hoverfly species [17]. Our results add further evidence that viruses traditionally considered ‘bee’ diseases are not restricted to Hymenoptera [11], and highlight the importance of understanding the role of non-bee pollinators in pathogen transmission. Interestingly, bee viruses were only detected in hoverfly species in the genus *Eristalis*, which mimic *A. mellifera* in both morphology and behaviour [18]. This presumed foraging niche overlap between *Eristalis* and *A. mellifera* may have increased the probability of exposure to bee pathogens via shared floral resources. By contrast, *Ep. balteatus* and *P. albimanus* are both generalist floral visitors that do not mimic bees [19].

Only viruses that were detected in co-foraging honeybees were detected in our hoverfly samples, and these were always at higher or equal prevalence in honeybees. Combined with high sequence similarity between isolates, this is consistent with spillover of these viruses into hoverflies, as has previously been suggested for bumblebees [4,5]. However, the detection of bee viruses in a sample does not imply infection and could be explained by vectoring. There was no evidence of viral replication for either BQCV or SBV in the hoverflies. But, given the low titres detected and subsequent likelihood of false negatives, we cannot rule out that these were true infections. While BQCV viral titres were much higher in honeybees, SBV titres in *Eristalis* were similar to those in honeybees, suggesting that *Eristalis* may potentially be acting as a host to SBV.

Regardless of whether hoverflies are active hosts or passive vectors of the pathogens [10], our results suggest that hoverfly flower visitors may play an important but previously unexplored role in pollinator disease networks. As abundant flower visitors sharing resources with both honeybees and wild bees, hoverflies may be capable of moving these pathogens around the landscape, facilitating transmission between susceptible bee species. *Eristalis tenax* is capable of extensive, long-distance migration [20], suggesting the potential for supra-national networks of pathogen transmission among pollinators. This is particularly concerning for emerging pathogens such as DWV-B, a recently discovered, highly virulent strain of the deformed wing virus [21]. Further work is now needed to investigate the role of hoverflies as both hosts and vectors for a wider range of pathogens, and the extent to which use of shared floral resources leads to spillover and transmission among species.

## Supplementary Material

First detection of bee viruses in hoverfly (syrphid) pollinators: Supplementary methods & results

## Data accessibility

Supporting datasets can be found in: https://doi.org/10.17637/rh.5706154 [22]. DNA sequences: Genbank accessions MG737448–MG737473.

## References

[1] Daszak P, Cunningham AA, Hyatt AD 2000 Emerging infectious diseases of wildlife—threats to biodiversity and human health. Science 287, 443–450. (10.1126/science.287.5452.443) 10642539

[2] Wilfert L, Long G, Leggett HC, Schmid-Hempel P, Butlin R, Martin SJ, Boots M 2016 Deformed wing virus is a recent global epidemic in honeybees driven by *Varroa* mites. Science 351, 594–597. (10.1126/science.aac9976) 26912700

[3] Dobson A 2004 Population dynamics of pathogens with multiple host species. Am. Nat. 164, S64–S78. (10.1086/424681) 15540143

[4] Fürst MA, McMahon DP, Osborne JL, Paxton RJ, Brown MJF 2014 Disease associations between honeybees and bumblebees as a threat to wild pollinators. Nature 364, 364–366. (10.1038/nature12977) PMC3985068 24553241

[5] McMahon DP, Fürst MA, Caspar J, Theodorou P, Brown MJF, Paxton RJ 2015 A sting in the spit: widespread cross-infection of multiple RNA viruses across wild and managed bees. J. Anim. Ecol. 84, 615–624. (10.1111/1365-2656.12345) 25646973 PMC4832299

[6] Gallai N, Salles J-M, Settele J, Vaissière BE 2009 Economic valuation of the vulnerability of world agriculture confronted with pollinator decline. Ecol. Econ. 68, 810–821. (10.1016/j.ecolecon.2008.06.014)

[7] Potts SG et al. 2016 Safeguarding pollinators and their values to human well-being. Nature 540, 220–229. (10.1038/nature20588) 27894123

[8] Tehel A, Brown MJF, Paxton RJ 2016 Impact of managed honey bee viruses on wild bees. Curr. Opin. Virol. 19, 16–22. (10.1016/j.coviro.2016.06.006) 27351468

[9] Durrer S, Schmid-Hempel P 1994 Shared use of flowers leads to horizontal pathogen transmission. Proc. R. Soc. Lond. B 258, 299–302. (10.1098/rspb.1994.0176)

[10] Graystock P, Goulson D, Hughes WOH 2015 Parasites in bloom: flowers aid dispersal and transmission of pollinator parasites within and between bee species. Proc. R. Soc. B 282, 20151371 (10.1098/rspb.2015.1371) PMC4632632 26246556

[11] Manley R, Boots M, Wilfert L 2015 Emerging viral disease risk to pollinating insects: ecological, evolutionary and anthropogenic factors. J. Appl. Ecol. 52, 331–340. (10.1111/1365-2664.12385) 25954053 PMC4415536

[12] Power EF, Stout JC 2011 Organic dairy farming: impacts on insect–flower interaction networks and pollination. J. Appl. Ecol. 48, 561–569. (10.1111/j.1365-2664.2010.01949.x)

[13] Rader R et al. 2016 Non-bee insects are important contributors to global crop pollination. Proc. Natl Acad. Sci. USA 113, 146–151. (10.1073/pnas.1517092112) 26621730 PMC4711867

[14] de Miranda JR et al. 2013 Standard methods for virus research in *Apis mellifera*. J. Apic. Res. 52, 1–56. (10.3896/IBRA.1.52.4.22)

[15] R Core Team. 2017 *R: A language and environment for statistical computing*. Vienna, Austria: R Foundation for Statistical Computing.

[16] Hothorn T, Hornik K, van de Wiel MA, Zeileis A 2006 A lego system for conditional inference. Am. Stat. 60, 257–263. (10.1198/000313006X118430)

[17] Evison SEF, Roberts KE, Laurenson L, Pietravalle S, Hui J, Biesmeijer JC, Smith JE, Budge G, Hughes WOH 2012 Pervasiveness of parasites in pollinators. PLoS ONE 7, e30641 (10.1371/journal.pone.0030641) 22347356 PMC3273957

[18] Golding YC, Edmunds M 2000 Behavioural mimicry of honeybees (*Apis mellifera*) by droneflies (Diptera: Syrphidae: *Eristalis* spp.). Proc. R. Soc. Lond. B 267, 903–909. (10.1098/rspb.2000.1088) PMC1690622 10853733

[19] Branquart E, Hemptinne J-L 2000 Selectivity in the exploitation of floral resources by hoverflies (Diptera: Syrphinae). Ecography 23, 732–742. (10.1111/j.1600-0587.2000.tb00316.x)

[20] Francuski L, Milankov V 2015 Assessing spatial population structure and heterogeneity in the dronefly. J. Zool. 297, 286–300. (10.1111/jzo.12278)

[21] McMahon DP, Natsopoulou ME, Doublet V, Fürst M, Weging S, Brown MJF, Gogol-Döring A, Paxton RJ 2016 Elevated virulence of an emerging viral genotype as a driver of honeybee loss. Proc. R. Soc. B 283, 20160811 (10.1098/rspb.2016.0811) PMC4936039 27358367

[22] Bailes EJ, Deutsch KR, Bagi J, Rondissone L, Brown MJF, Lewis OT 2018 Data from: First detection of bee viruses in hoverfly (syrphid) pollinators *Dryad Digital Repository*. (10.17637/rh.5706154) PMC5830674 29491032

