# Supplementary material for: First detection of bee viruses in hoverfly (syrphid) pollinators
Source: Biol Lett. 2018 Feb 28;14(2):20180001. doi: 10.1098/rsbl.2018.0001 (PMC5830674; doi:10.1098/rsbl.2018.0001)
Supplement: First detection of bee viruses in hoverfly (syrphid) pollinators: Supplementary methods & results [file rsbl20180001supp1.docx]

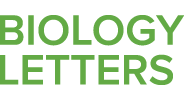


**First detection of honey bee viruses in hoverfly (syrphid) pollinators**

Supplementary information

Emily J Bailes^1*^, Kaitlin R Deutsch^1,2,3,4*^, Judit Bagi^1^, Lucila Rondissone^1^, Mark J F Brown^1†^, Owen T Lewis^2†^

^1^School of Biological Sciences, Royal Holloway University of London, Egham TW20 0EX, UK

^2^School of Geography and the Environment, University of Oxford, South Parks Road, Oxford, OX1 3QY, UK

^3^Department of Zoology, University of Oxford, South Parks Road, Oxford OX1 3PS, UK

^4^Department of Entomology, Cornell University, 2130 Comstock Hall, Ithaca, NY 14853

*These authors contributed equally to the work

†Equal senior authors

Corresponding author: Emily J Bailes [emilyjbailes@gmail.com](mailto:ebailesjbailes@gmail.com)

**Supplementary Methods**

**Sample collection**

Honey bees and four of the most common UK species of hoverfly (*Episyrphus balteatus, Platycheirus albimanus, Eristalis tenax, and Eristalis arbustorum*) were collected 16-22 July 2016 from grassland and open woodland habitats at Wytham Woods in Oxfordshire, United Kingdom (51.77°N, -1.33°W). Verbal permission was obtained for collecting specimens at Wytham Woods, a tract of land owned by the University of Oxford. None of the focal species have endangered or protected status. The nearest managed honey bee colonies were approximately 1.5 km from the study site (N. Fisher, personal communication). Within Wytham, honey bees and *Eristalis* species were collected while foraging on flowers in open meadows (most commonly wild parsnip, *Pastinaca sativa*), and the majority of *P. albimanus and Ep. balteatus* were collected from wooded tracks and shaded, grassy margins along the roadside. Twenty individuals of each species were collected for screening. Only females were collected for both *A. mellifera* and *P. albimanus*, as only female honey bees forage on flowers, and male *P. albimanus* are morphologically indistinguishable from other *Platycheirus* species. Due to higher numbers of male foragers at the time of collection, for *Er. tenax*, 17 males and 3 females were collected, and for *Er. arbustorum,* 15 males and 5 females were collected, while for *Ep. balteatus* 10 males and 10 females were collected. All individuals were brought into the lab alive and identified under the scope if necessary, and stored immediately at -80°C to prevent RNA degradation.

**RNA extraction & cDNA synthesis**

Bee and hoverfly abdomens were dissected on dry ice, submerged in 600µL Tri-Reagent buffer and homogenized in a TissueLyser II (Qiagen) with 4 mm (diameter) chrome-plated steel balls (Atlas Ball and Bearing Company Ltd, Walsall, UK) for 4 minutes at 30 Hz. Homogenized samples were centrifuged for 15 minutes at 12,000 rpm, 4°C. Total RNA was then extracted using a Direct-zol™ RNA MiniPrep kit (Zymo Research, California, USA) following the manufacturer’s protocol with the exception that the on-column DNA digestion was not carried out. Samples were eluted in 30µL RNase/DNase-free water. The concentration of RNA was determined using a NanoDrop. Total complementary DNA (cDNA) was synthesized from 2µg of the RNA template in a 25 µl reaction using M-MLV Reverse Transcriptase (Promega) with 0.5 µg random hexamers (Invitrogen), following the manufacturer’s protocol.

**Virus screening by RT-PCR**

Presence or absence of six common bee viruses (Acute Bee Paralysis virus ABPV, Black Queen Cell Virus BQCV, Deformed Wing Virus strain A DWV-A, and strain B DWV-B, Slow Bee Paralysis Virus SBPV, and SacBrood Virus SBV) was determined by RT-PCR. Total reaction volume was 20 µl, using 0.5 U of GoTaq G2 flexi polymerase (Promega), with 2.5 µl of template (0.1x cDNA), 1x reaction buffer, 2.5 mM MgCl_2_, 0.2 µM dNTPs (each), and 0.25 µM primers (each). Samples were amplified at 95 °C for 2 minutes, followed by 37 cycles of 95 °C for 30 seconds, T_a_ for 30 seconds, and 72 °C for 1 minute, and a final extension step of 72°C for 5 minutes (T_a_ and primer sequences are given in Table S1). Positive and negative controls were included in each PCR run. PCR products were visualized under UV light on a 1.5% agarose gel stained with 0.3 µg/ml ethidium bromide.

Positive samples identified by the amplification of the correct-sized product were verified by amplification in an independent RT-PCR reaction and subsequent Sanger sequencing (by Source Bioscience, Cambridge) of the PCR product following clean up (using Promega Wizard® SV Gel and PCR Clean-Up System) to confirm they mapped to the virus of interest in the National Center for Biotechnology Information (NCBI) database.

**Quantification of viral titres by qRT-PCR**

## Viral titres were quantified for SBV and BQCV (the viruses we detected most frequently) for all samples identified as positive for these viruses by qRT-PCR (primers in Table S1). For absolute quantification, triplicate qRT-PCR was carried out with a Roche LightCycler® 480 II, using LightCycler^®^ 480 SYBR Green I (Roche) mastermix with a 10 µl final reaction volume, 0.5 µM primers (each) and 2 µl of template (0.2x cDNA). A standard curve ranging from 2 x 10^8^ to 2 x 10^0^ genome equivalents for SBV and 2 x 10^8^ to 2 x 10^1^ genome equivalents for BQCV was contained in each reaction run. Standards were generated by cloning a PCR fragment from an *Apis* virus-positive sample into the pGEM t-easy (Promega) vector. Thermocycler conditions were as follows: 5 min at 95°C, followed by 45 cycles of 10s at 95°C, 10s at T_a_°C and 15s at 72°C (read). Following PCR, DNA was denatured for 5s at 95 °C and cooled to 65 °C for 1 min. A melting profile was generated from 65 to 97 °C (0.11°C per second increments) to rule out false positives. A no-template control of water was included in each reaction run. Quantification was based on the standard curve calculated on the same run. Primer efficiencies were 97% and 102% for BQCV and SBV, respectively.

**Strand-specific RT-PCR**

To detect the negative strand of SBV and BQCV, indicative of virus replication, the protocol of de Miranda et al. [1; section 10.2.8.1] was followed using Superscript III (Invitrogen). Tagged primers are detailed in Table S1. A combined exonuclease and restriction digest was carried out on the tagged cDNA to remove excess primers (which can cause false positives) and reduce non-specific priming during PCR respectively. A final reaction volume of 20 µl, containing 5 µl cDNA, 20 U endonuclease I (ThermoScientific), 10 U EcoRV (Promega), 10 U Bgl I (Promega), 0.1 µg/µl BSA, and 0.75 X Buffer D (accounting for the salts in the cDNA buffer; Promega), was incubated at 37 ºC for 30 min and then denatured at 80 ºC for 15 min. A 0.2 X dilution of the digestion was used as template in RT-PCR, carried out (as above) with the tag and virus-specific primers. Negative controls of the strand-specific cDNA template with only the forward or reverse primer were used to test for non-specific priming and efficient removal of residual cDNA synthesis primers that could result in false positives. For replication-positive samples, a second reverse transcription of the sample was carried out without virus-specific (or any other) primers to check for false positives resulting from the self-priming of cDNA synthesis.

**Sequence similarity**

We determined the level of nucleotide identity between the SBV and BQCV sequences that we amplified from this study using multiple sequence alignment tool Clustal Omega v1.2.4

(<https://www.ebi.ac.uk/Tools/msa/clustalo/>). Values are taken from the ‘Percent Identity Matrix’, which makes a conservative calculation where ambiguous bases are not identical to their components, eg. N is not identical to A, T, C, or G; W is not identical to A or T; but N is identical to N etc. We compared a 345 bp overlapping section of high quality sequence from the SBV capsid gene and 696 bp section of BQCV RNA-dependent RNA polymerase gene. However, for one SBV sequence from *Er. tenax* (MG737464) we were only able to obtain a 142 bp section of overlapping good quality sequence despite multiple sequencing attempts. Therefore for this sequence, %identity was calculated for the 142 bp high quality region only. Similarly, the first 34 bp of one BQCV sequence from *A. mellifera* (MG737459) was not included in analyses of BQCV sequence identity because its quality was assessed as low.

**Table S1 -** Primer pairs and RT-PCR conditions used during this study

| **Virus** | **Purpose** | **Primer name** | **Primer sequence** | **T_a_** | **Reference** | **Product size** |
| --- | --- | --- | --- | --- | --- | --- |
| **ABPV** | RT-PCR screen | ABPV_F5088 | CYATGGACACACCCTATGTG | 55 ºC | [2] | 1034bp |
|  |  | ABPV_R6122 | CGCCATTTTGGTACTTCTCC |  |  |  |
| **BQCV** | RT-PCR screen | BQCV_F4119 | TCCYCCAGTTCAACCATCTA | 60 ºC | [2] | 1257bp |
|  |  | BQCV_R5376 | AACGTTGCCTAGRTTCGTCA |  |  |  |
| **DWV-A** | RT-PCR screen | DWV-F7993 | AACTGGCGAYCATACTCAGC | 57 ºC | [3] | 644bp |
|  |  | DWV-8577R | WCCAGGCACMCCACATACAG |  |  |  |
| **DWV-A** | RT-PCR screen | DWV for (4241) | GATGGTCCGCGGCTAAGA | 54 ºC | [4] | 759bp |
|  |  | DWV rev (4980) | CGGCAGATATAACAGTACTTG |  |  |  |
| **DWV-B** | RT-PCR screen | 152 (F) | CTGTAGTTAAGCGGTTATTAGAA | 55 ºC | [5] | 1428bp |
|  |  | 154 (R) | CTGAAGTACTAATCTCTGAG |  |  |  |
| **DWV-B** | RT-PCR screen | VDV for (5520) | CATGGAAATGGGATCAAACC | 56 ºC | [4] | 722bp |
|  |  | VDV rev (6180) | CTTCCAAGGGCTCATCCATA |  |  |  |
| **SBV** | RT-PCR screen | SBV-VP1b-F | GCACGTTTAATTGGGGATCA | 55 ºC | [6] | 693bp |
|  |  | SBV-VP1b-R | CAGGTTGTCCCTTACCTCCA |  |  |  |
| **SBPV** | RT-PCR screen | SBPV_9_774F | GAGATGGATMGRCCTGAAGG | 55 ºC | [2] | 915bp |
|  |  | SBPV_9_1689R | CATGAGCCCAKGARTGTGAA |  |  |  |
| **BQCV** | qRT-PCR | qBQCV2-F | GGAGTCGCAGAGTTCCAAATA | 57 ºC | [7] | 210bp |
|  |  | qBQCV2-R | GAGATGCGTGAATACAGGGC |  |  |  |
| **SBV** | qRT-PCR | SBV-F434 | AACGTCCACTACACCGAAATGTC | 60 ºC | [8] | 70 bp |
|  |  | SBV-R503 | ACACTGCGCGTCTAACATTCC |  |  |  |
| **BQCV** | negative strand specific cDNA synthesis | Tag-BQCV-sense | agcctgcgcaccgtggTCAGGTCGGAATAATCTCGA | na | [9] | na |
| **general** | negative strand specific RT-PCR | tag | AGCCTGCGCACCGTGG | na | [9] | na |
| **BQCV** | negative strand specific RT-PCR | BQCV-antisense | GCAACAAGAAGAAACGTAAACCAC | 55 ºC | [9] | 420bp (with tag) |
| **SBV** | negative strand specific cDNA synthesis | Tag-SB7f | agcctgcgcaccgtggGGAGATGTTAGAAATACCAACCGATTCC | na | [10] | na |
| **SBV** | negative strand specific RT-PCR | SB8R | CCATTAAACAAATCGGTATAAGAGTCCACT | 57 ºC | [10] | 200bp |
| **SBV** | qRT-PCR standard | SB1-f | ACCAACCGATTCCTCAGTAG | 58 ºC | [11] | 487bp |
|  |  | SB2-r | CCTTGGAACTCTGCTGTGTA |  |  |  |

**Supplementary results**

**False negatives for virus presence and non-specific amplification of primers**

*SBV*

All samples that were initially identified as positive for SBV were later confirmed to contain SBV through Sanger sequencing and/or qRT-PCR.

*ABPV*

Following the intial detection of ABPV in two *A. mellifera* workers and 3 hoverflies we subsequently confirmed the presence of ABPV in only a single sample originating from *A. mellifera.* The remaining samples were excluded either because the product could not be amplified in an independent PCR reaction (one *A. mellifera*, one hoverfly) or because when re-amplified the product size was incorrect.

*BQCV*

Following the initial detection of BQCV in four *Er. tenax* samples, only two of these could not be re-amplified, including by qRT-PCR. For *A. mellifera* and *Er. arbustorum,* all samples initially identified were confirmed to be present by Sanger Sequencing and/or qRT-PCR.

*DWV complex*

For the DWV complex we found the results from hoverfly samples to be highly inconsistent for most sets of primers and we were unable to verify the presence of DWV-A in our samples following the use of two different primer sets. This was largely due to spurious amplification in the majority of hoverfly (but not *A. mellifera*) samples, regardless of whether a product of the expected size was amplified (Fig S1). We had similar problems with DWV-B specific primer set VDV for (5520)/ VDV rev (6180). However primer set 152 (F)/154 (R), which is specific to DWV-B, non-specific amplification did not occur and we were able to verify the presence of this virus in 7/20 *A. mellifera* samples and one hoverfly individual (*Er. tennax*) following a nested PCR with internal primers VDV for (5520)/ VDV rev (6180). This non-specific binding in non-*Apis* samples suggests that these primers are binding to DWV-like sequence in the hoverfly genome.

*SBPV*

SBPV was not detected in any of our samples.


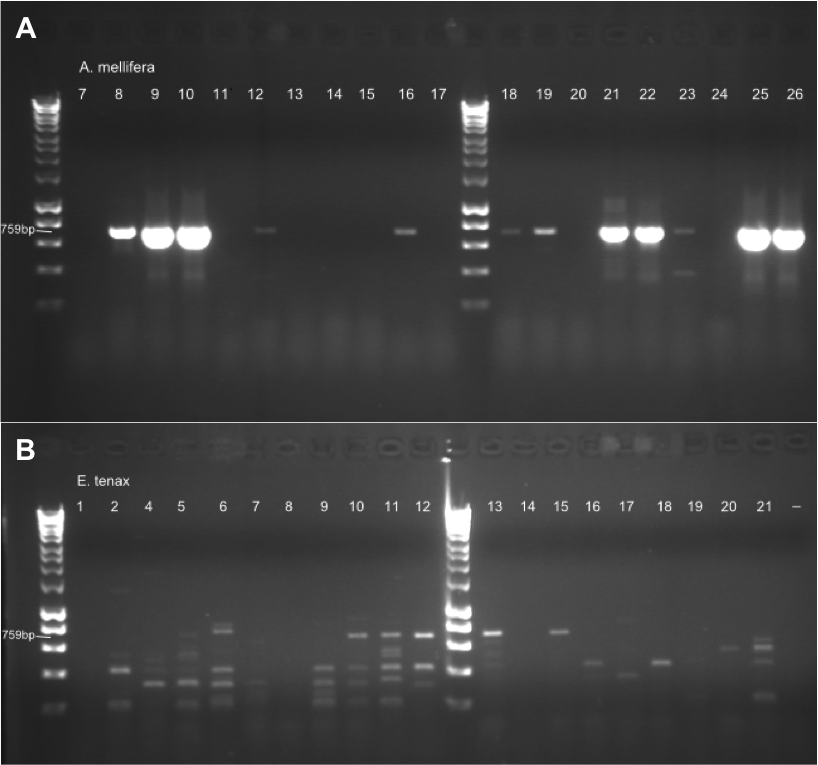


**Figure S1 –** An example of spurious amplification in hoverfly samples (*Er. tenax*; B) vs. *A. mellifera* (A) samples for primers amplifying the DWV complex. In this example the primers VDV for (5520)/VDV rev (6180) were used (expected product size 759bp). Samples are from the same PCR run. A subsequent independent PCR of the *Er. tenax* samples 10, 11, 12, 13 and 15 with the correct product size in the above PCR could not re-amplify the correct product size or enough product to gel extract for sequencing.

**Table S2 -** The NCBI ID for sequences generated from this study and their relationship to sequences currently in the NCBI database.

| Sample ID | Species | Virus | NCBI Seq ID | Closest NCBI sequence & description | nt identity/coverage |
| --- | --- | --- | --- | --- | --- |
| Am7 | *A. mellifera* | SBV | MG737464 | HG764799.1  Sacbrood virus partial CP gene for capsid protein, isolate Av from *Andrena vaga* | 95%/100% |
| Am8 | *A. mellifera* | BQCV | MG737455 | EF517515.1  Black queen cell virus strain HUNGARY-10 non-struct polyprot and struct polyprot genes, compl cds | 94%/100% |
| Am9 | *A. mellifera* | BQCV | MG737456 | KY243932.1  Black queen cell virus strain PP, complete genome | 89%/100% |
| Am10 | *A. mellifera* | SBV | MG737465 | HG764798.1  SBV partial CP gene from *A. mellifera* | 97%/98% |
| Am10 | *A. mellifera* | DWV-B | MG737449 | AY251269.2  Varroa destructor virus 1, complete genome | 99%/100% |
| Am12 | *A. mellifera* | SBV | MG737466 | HG764799.1  Sacbrood virus partial CP gene for capsid protein, isolate Av from *Andrena vaga* | 96%/95% |
| Am16 | *A. mellifera* | DWV-B | MG737450 | AY251269.2  Varroa destructor virus 1, complete genome | 100%/100% |
| Am19 | *A. mellifera* | BQCV | MG737457 | EF517515.1  Black queen cell virus strain HUNGARY-10 non-struct polyprot and struct polyprot genes, compl cds | 93/100% |
| Am20 | *A. mellifera* | ABPV | MG737448 | AF486072.2  Acute bee paralysis virus isolate Hungary 1, complete genome | 97%/100% |
| Am20 | *A. mellifera* | SBV | MG737467 | Sacbrood virus partial CP gene for capsid protein, isolate 009 from *Apis mellifera* | 97%/98% |
| Am21 | *A. mellifera* | DWV-B | MG737451 | AY251269.2  Varroa destructor virus 1, complete genome | 99%/100% |
| Am22 | *A. mellifera* | DWV-B | MG737452 | AY251269.2  Varroa destructor virus 1, complete genome | 100%/100% |
| Am24 | *A. mellifera* | BQCV | MG737458 | KY243932.1  Black queen cell virus strain PP, complete genome | 90%/100% |
| Am26 | *A. mellifera* | BQCV | MG737459 | KY243932.1  Black queen cell virus strain PP, complete genome | 88%/100% |
| Am26 | *A. mellifera* | DWV-B | MG737453 | AY251269.2  Varroa destructor virus 1, complete genome | 99%/100% |
| Am26 | *A. mellifera* | SBV | MG737468 | HG764798.1  Sacbrood virus partial CP gene for capsid protein, isolate 009 from *Apis mellifera* | 97%/95% |
| Ea3 | *Er. arbustorum* | DWV-B | MG737454 | AY251269.2  Varroa destructor virus 1, complete genome | 99%/100% |
| Ea14 | *Er. arbustorum* | BQCV | MG737460 | EF517515.1  Black queen cell virus strain HUNGARY-10 non-struct polyprot and struct polyprot genes, compl cds | 94%/99% |
| Ea20 | *Er. arbustorum* | BQCV | MG737461 | KY243932.1  Black queen cell virus strain PP, complete genome | 89%/99% |
| Ea20 | *Er. arbustorum* | SBV | MG737469 | HG764798.1  Sacbrood virus partial CP gene for capsid protein, isolate 009 from *Apis mellifera* | 96%/98% |
| Et12 | *Er. tenax* | BQCV | MG737462 | KY243932.1  Black queen cell virus strain PP, complete genome | 89%/100% |
| Et13 | *Er. tenax* | SBV | MG737470 | HG764798.1  Sacbrood virus partial CP gene for capsid protein, isolate 009 from *Apis mellifera* | 97%/100% |
| Et22 | *Er. tenax* | SBV | MG737471 | HG764798.1  Sacbrood virus partial CP gene for capsid protein, isolate 009 from *Apis mellifera* | 96%/98% |
| Et5 | *Er. tenax* | SBV | MG737472 | HG764798.1  Sacbrood virus partial CP gene for capsid protein, isolate 009 from *Apis mellifera* | 96%/98% |
| Et6 | *Er. tenax* | BQCV | MG737463 | KY243932.1  Black queen cell virus strain PP, complete genome | 89%/100% |
| Et6 | *Er. tenax* | SBV | MG737473 | HG764798.1  Sacbrood virus partial CP gene for capsid protein, isolate 009 from *Apis mellifera* | 96%/98% |

| Sample ID | Am7 | Ea20 | Am20 | Et6 | Am26 | Am10 | Et5 | Et22 | Am12 |
| --- | --- | --- | --- | --- | --- | --- | --- | --- | --- |
| Am7 | - |  |  |  |  |  |  |  |  |
| Ea20 | 95.1 | - |  |  |  |  |  |  |  |
| Am20 | 95.1 | 98.8 | - |  |  |  |  |  |  |
| Et6 | 95.1 | 99.1 | 99.4 | - |  |  |  |  |  |
| Am26 | 96.5 | 97.4 | 97.4 | 97.7 | - |  |  |  |  |
| Am10 | 97.9 | 97.1 | 97.1 | 97.4 | 98.6 | - |  |  |  |
| Et5 | 96.5 | 96.2 | 96.2 | 96.5 | 97.7 | 98.6 | - |  |  |
| Et22 | 96.5 | 96.2 | 96.2 | 96.5 | 97.7 | 98.6 | 99.7 | - |  |
| Am12 | 97.9 | 96.2 | 96.2 | 96.5 | 97.7 | 99.1 | 98.8 | 98.6 | - |

**Table S3 –** The nucleotide similarity between SBV sequences from *A. mellifera* (identified by Am##), *Er. tenax* (identified by Et##) and *Er. arbustorum* (Ea20). Matrix generated using CLUSTAL Omega (1.2.4) multiple sequence alignment

| Sample ID | Am19 | Am8 | Ea14 | Am26 | Ea20 | Et12 | Am9 | Am24 | Et6 |
| --- | --- | --- | --- | --- | --- | --- | --- | --- | --- |
| Am19 | - |  |  |  |  |  |  |  |  |
| Am8 | 96.8 | - |  |  |  |  |  |  |  |
| Ea14 | 97.3 | 98.6 | - |  |  |  |  |  |  |
| Am26 | 89.0 | 89.1 | 88.8 | - |  |  |  |  |  |
| Ea20 | 86.6 | 86.9 | 86.6 | 93.2 | - |  |  |  |  |
| Et12 | 87.4 | 87.8 | 87.5 | 93.8 | 98.9 | - |  |  |  |
| Am9 | 87.4 | 87.6 | 87.4 | 94.0 | 99.0 | 99.6 | - |  |  |
| Am24 | 87.5 | 87.8 | 87.5 | 94.1 | 99.1 | 99.7 | 99.9 | - |  |
| Et6 | 87.5 | 87.8 | 87.5 | 94.1 | 99.1 | 99.7 | 99.9 | 100.0 | - |

**Table S4** - The nucleotide similarity between BQCV sequences from *A. mellifera* (identified by Am##), *Er. tenax* (identified by Et##) and *Er. arbustorum* (Ea20). Matrix generated using CLUSTAL Omega (1.2.4) multiple sequence alignment

**References**

1. de Miranda JR *et al.* 2013 Standard methods for virus research in *Apis mellifera*. *J. Apic. Res.* **52**, 1–56. (doi:10.3896/IBRA.1.52.4.22)

2. Manley R, Boots M, Wilfert L. 2017 Condition - dependent virulence of slow bee paralysis virus in *Bombus terrestris* : are the impacts of honeybee viruses in wild pollinators underestimated ? *Oecologia* **184**, 305–315. (doi:10.1007/s00442-017-3851-2)

3. Wilfert L, Long G, C LH, Schmid-Hempel P, Butlin R, Martin SJ, Boots M. 2016 Deformed wing virus is a recent global epidemic in honeybees driven by *Varroa* mites. **351**, 594–597. (doi:10.1126/science.aac9976)

4. Zioni N, Soroker V, Chejanovsky N. 2011 Replication of Varroa destructor virus 1 ( VDV-1 ) and a Varroa destructor virus 1 – deformed wing virus recombinant ( VDV-1 – DWV ) in the head of the honey bee. *Virology* **417**, 106–112. (doi:10.1016/j.virol.2011.05.009)

5. Ryabov E V, Wood GR, Fannon JM, Moore JD, Bull JC, Chandler D, Mead A, Burroughs N, Evans DJ. 2014 A virulent strain of Deformed wing virus ( DWV ) of honeybees ( *Apis mellifera* ) prevails after *Varroa destructor*-mediated , or in vitro , transmission. *PLoS Pathog.* **10**, e1004230. (doi:10.1371/journal.ppat.1004230)

6. Singh R *et al.* 2010 RNA viruses in Hymenopteran pollinators: evidence of inter-taxa virus transmission via pollen and potential impact on non-Apis Hymenopteran species. *PLoS One* **5**, e14357. (doi:10.1371/journal.pone.0014357)

7. Choi NR, Jung C, Lee DW. 2015 Optimization of detection of black queen cell virus from *Bombus terrestris* via real-time PCR. *J. Asia. Pac. Entomol.* **18**, 9–12. (doi:10.1016/j.aspen.2014.10.010)

8. Blanchard P *et al.* 2014 Development and validation of a real-time two-step RT-qPCR TaqMan ® assay for quantitation of Sacbrood virus ( SBV ) and its application to a field survey of symptomatic honey bee colonies. *J. Virol. Methods* **197**, 7–13. (doi:10.1016/j.jviromet.2013.09.012)

9. Peng W *et al.* 2011 Host range expansion of honey bee Black Queen Cell Virus in the bumble bee , *Bombus huntii*. *Apidologie* **42**, 650–658. (doi:10.1007/s13592-011-0061-5)

10. Gong HR, Chen XX, Chen YP, Hu FL, Zhang JL, Lin ZG, Yu JW, Zheng HQ. 2016 Evidence of *Apis cerana* Sacbrood virus infection in *Apis mellifera*. *Appl. Environ. Microbiol.* **82**, 2256–2262. (doi:10.1128/AEM.03292-15)

11. Grabensteiner E *et al.* 2001 Sacbrood virus of the honeybee ( *Apis mellifera* ): rapid identification and phylogenetic analysis using reverse transcription-PCR. *Clin. Diagn. Lab. Immunol.* **8**, 93–104. (doi:10.1128/CDLI.8.1.93)
